# Isolation and Characterization of Microsatellite Loci for Stys's Bush-Cricket, *Isophya stysi*, and Cross-Species Amplification in Closely Related Species from the Phaneropteridae Family

**DOI:** 10.1673/031.013.5501

**Published:** 2013-06-14

**Authors:** Elena I. Iorgu, Oana P. Popa, Ana-Maria Krapal, Luis O. Popa

**Affiliations:** 1 “Grigore Antipa” National Museum of Natural History, Molecular Biology Department, Bucharest, RO 01 1341, Romania; 2 Departments of Biochemistry and Molecular Biology, University of Bucharest, Bucharest, RO 050095, Romania; 3 Faculty of Biology, Alexandru loan Cuza University of lasi, laşi, RO 700505, Romania

**Keywords:** Orthoptera, polymorphic, population genetics

## Abstract

Ten microsatellite loci were isolated and characterized for Stys's bush-cricket, *Isophya stysi* Cejchan (Orthoptera: Tettigoniidae), an endemic Orthoptera species to the Carpathian Basin, using an enriched genomic library procedure. The polymorphism of these loci were tested in two populations of *I. stysi*, and the number of alleles per locus varied from 4 to 16. The expected and observed heterozygosities ranged from 0.612 to 0.925 and from 0.625 to 1.000, respectively. The interspecific applicability of these microsatellites was evaluated by amplification in 20 related species: *Isophya camptoxypha, Isophya sicula, Isophya ciucasi, Isophya pienensis, Isophya harzi, Isophya kraussii, Isophya zubovskii, Isophya rectipennis, Isophya modesta, Isophya longicaudata, Isophya dobrogensis, Isophya hospodar, Isophya speciosa, Isophya modestior, Poecilimon fussii, Poecilimon affinis, Polysarcus denticauda, Barbitistes constrictus, Leptophyes discoidalis, Phaneroptera falcata*. All primer pairs for the 10 loci yielded successful amplifications in at least one other taxon from the *Isophya* genus. This set of microsatellite loci would be useful for genetic studies in *I. stysi* and other species of the genus *Isophya*.

## Introduction

The genus *Isophya* (Orthoptera: Tettigoniidae) is one of the largest of the Palearctic Orthoptera, comprising a large number of species with a high degree of morphological similarity (Heller 1988; Warchałowska-Śliwa et al. 2008; Chobanov 2009; Grzywacz-Gibała et al. 2010). The genus has a high number of endemic species (Sevgili 2003; Sevgili and Heller 2003), most of them having restricted distribution ranges, limited mobility, and specific topographic requirements (Sevgili et al. 2006). Many European *Isophya* species are found in isolated populations with low densities of individuals, displaying habitat dependence due to their feeding preferences for dicotyledonous plants (Bauer and Kenyeres 2006). These characteristics make them vulnerable to anthropogenic disturbances that lead to the reduction of their natural habitat, and many of the species have been rendered endangered.

*Isophya stysi* Cejchan lives in small populations throughout its range and has a fragmented distribution (Orci et al. 2005). It lives in mesophylic grasslands, near forests and forest clearings (Kis 1960; Pecsenye et al. 2003), and adults can be found mainly on high herbaceous plants and small shrubs (Iorgu and Iorgu 2008). The species is endemic to the Carpathian Basin (Kis 1960; Nagy 2005), being described from the Carpathian Mountains in Slovakia, and is also found in eastern Hungary, Poland, Romania, and Ukraine (Heller et al. 2004). In Romania, it is common in the Apuseni Mountains and Transylvania, and rare in the Oriental Carpathians and in the forests of the Moldavian Plateau (Iorgu et al. 2008).

*I. stysi* is protected by national and European laws (present on Annex II of EU Habitat Directive) and requires strict conservation measures. In order to ensure effective conservation management, the genetic diversity of the populations of this endangered species needs to be evaluated. Microsatellite DNA is an optimal molecular marker for studies of genetic diversity in natural populations as it can assess population structure (Goldstein and Schi öfterer 1999). Population genetic analyses can also be used to identify management units based on ecological and genetic variation, and to trace threatened populations in need of conservation priority (Wan et al. 2004; Palsbøll et al. 2007). The major aim of this study was to describe the first microsatellite loci for *I. stysi* and to report the results of cross-species amplification tests in 20 other related Orthoptera species.

## Materials and Methods

The isolation of the microsatellite loci for *I. stysi* was performed following a standard protocol for the construction of a microsatellite-enriched library (Bloor et al. 2001). Genomic DNA was isolated from the hind femurs of two individuals of *I. stysi* using a phenolclorophorm protocol (Sokolov 2000).

Approximately 10 µg of genomic DNA was digested using *Sau*3AI restriction enzyme (Fermentas UAB, www.fermentas.lt). Adaptor-ligated DNA fragments ranging from 400 to 1000 bp were selected, and enriched using 3′ biotin-labelled CA and GA repeat oligos bound to streptavidin coated magnetic beads (M-280 Dyneabeads, Dynal, Invitrogen, www.invitrogen.com). The DNA fragments were then ligated into the pJET 1.2 vector (Fermentas UAB) and transformed into DH5α *Escherichia coli* competent cells for cloning. The enriched genomic library was screened for repetitive sequences, and 73 clones containing inserts with microsatellite motives were selected. These were further sequenced using the LICOR 4300L Genetic Analyzer. The similarity of the flanking regions and the microsatellite length were determined with SciRoKo 3.4 (Kofler et al. 2007), and 25 sequences were chosen suitable for primer design. Primers were designed using Primer 3 program (Rozen and Skaletsky 2000).

All 25 primer pairs were initially tested for amplification using 8 individuals of *I. stysi*. Primers that yielded products of expected sizes were given an M13 sequence tail to allow analysis on the LICOR 4300L genetic analyzer (M13F: 5′-cacgacgttgtaaaacgac-3′, M13R: 5′ -ggataacaatttcacacagg-3′).

The genotyping reactions were performed in a 10 µL reaction volume, containing about 30 ng of DNA template, 10 mM Tris-HCl (pH 8.8 at 25° C), 50 mM KCl, 0.08% (v/v) Nonidet P40, 1.5 to 2.5 mM MgCl_2_ (see Table 1 for details for each locus), each dNTP at 0.1 mM, each primer at 0.1 µM, 0.02 µM of IRD700, or IRD800 labeled M13 primers (the same sequence as the M13 tails), and 0.5 units of Taq DNA polymerase (Fermentas UAB).

The PCR program used consisted of 5 minutes denaturating at 95° C, followed by 30 cycles of 30 sec at 95° C, 30 sec at the annealing temperature (see Table 1 for each locus), and 45 sec at 72° C, ending with a 7 min final elongation stage at 72° C. The genotyping process was performed using the Saga^GT^ 3.2 software package (LI-COR Biosciences, www.licor.com).

The degree of polymorphism of the 10 selected loci was tested in two populations of *I. stysi* collected from two mesophylic meadows in Romania (23 individuals from CeahlăuMountain, Neamţ, County: 47° 01′ 14′ ‘N, 25° 57’ 16″ E; 21 individuals from Nucşoara, Alba County: 45° 29′ 09″ N, 22° 56′ 03″ E). Genomic DNA was extracted from the middle leg of each individual using the Nucleospin Tissue kit (Macherey-Nagel, www.mnnet.com).

The null alleles frequencies were estimated by a maximum likelihood algorithm as implemented in FreeNA (Chapuis and Estoup 2004; Chapuis et al 2008), and tests for linkage disequilibrium were carried out using GenePop v. 4.0.10 (Raymond and Rousset 1995; Rousset 2008).

## Results and Discussion

Only ten out of 25 primer pairs proved to be polymorphic and were deemed acceptable for population genetic studies. The number of alleles at each polymorphic locus, their size ranges, observed and expected heterozygosities, as well as deviation from the Hardy-Weinberg equilibrium were calculated using GenAlEx 6.4 (Peakall and Smouse 2006) and are summarized in Table 1. The microsatellite loci showed high levels of polymorphism, the number of alleles per locus ranging from 4 to 13 in the population from Ceahlau Mountain and from 4 to 16 in the population from Nuc§oa-ra. In the population from Ceahlau Mountain, the observed and expected heterozygosities ranged from 0.625 to 0.957 and from 0.612 to 0.891 respectively, with an average of 0.843 and 0.799 respectively. In the population from Nuc-§oa-ra the observed and expected heterozygosities ranged from 0.714 to 1 and from 0.684 to 0.925 respectively, with an average of 0.876 and 0.844 respectively.

Significant deviation from the HardyWeinberg equilibrium was observed in 3 out of 20 possible single exact locus tests (*p* < 0.05), IST3 only in the Ceahlau Mountain population, and 1ST 18 and 1ST 23 only in the Nuc-§oa-ra population. Null alleles were estimated as present in IST15 (estimated frequency f.e. = 0.028), IST21 (f.e. = 0.059), and IST24 (f.e. = 0.019) loci in the Ceahlau Mountain population, and in IST23 locus (f.e. = 0.073) in the Nuc-§oa-ra population. These results, together with the relative small sample size (Ceahlaua: 23; Nuc-§oa-ra: 21), may explain the deviation observed in some of the Hardy-Weinberg equilibrium tests. No significant linkage disequilibrium was found between loci pairs in tests performed across all populations.

The molecular variance analysis, calculated using GenAlEx 6.4, showed significant differentiation between the two populations (*p* = 0.01), with a moderate pairwise FST value of 0.056. The genetic differentiation between the two populations can be explained by the geographical distance between them (almost 290 km in a straight line), which can determine a low level of geneflow.

In order to assess interspecific amplification, the polymorphic loci were also tested in 20 additional species from the Phaeropteridae family: 14 species of the genus *Isophya* (*I. camptoxypha, I. sicula, I. ciucasi, I. pienensis, I. harzi, I. kraussii, I. zubovskii, I. rectipennis, I. modesta, I. longicaudata, I. dobrogensis, I. hospodar, I. speciosa, I. modestior*), two species of the genus *Poecilimon (P. fussii, P. affinis*), and four species from different genera (*Barbitistes constrictus, Polysarcus denticaudus, Leptophyes discoidalis*, and *Phaneroptera nana*) (Table 2). DNA samples from two individuals of each species were genotyped using the same PCR conditions used for *I. stysi*. All primer pairs amplified in at least one other taxon from the *Isophya* genus, and only 6 of them amplified for species outside of the genus (IST2, IST5, IST6, IST9, IST15, and IST21). IST2 and IST5 loci amplified in all *Isophya* species tested and IST15 locus amplified in 12 species. Eight of the microsatellite loci (Table 2) amplified in *I. modestior*, which is considered closely related to *I. stysi* from morphological and bioacustical data (Warchałowska-Śliwa et al. 2008).

These data show that the microsatellite markers isolated for *I. stysi* may prove to be very useful in population genetic studies on other species of the genus *Isophya*, but their potential for cross-species amplification is limited outside the genus. These novel polymorphic loci should be a useful tool to study the genetic diversity and structure of *I. stysi* populations and to develop better conservation measures for this endangered species.

**Table 1.**
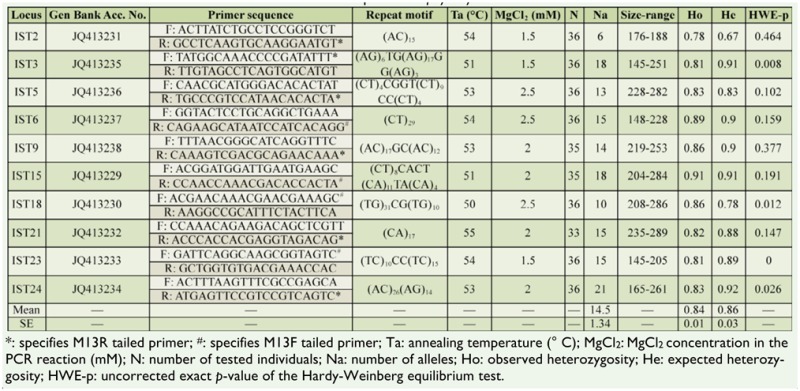
Characterization of ten microsatellite loci developed for *Isophya stysi*.

**Table 2.**
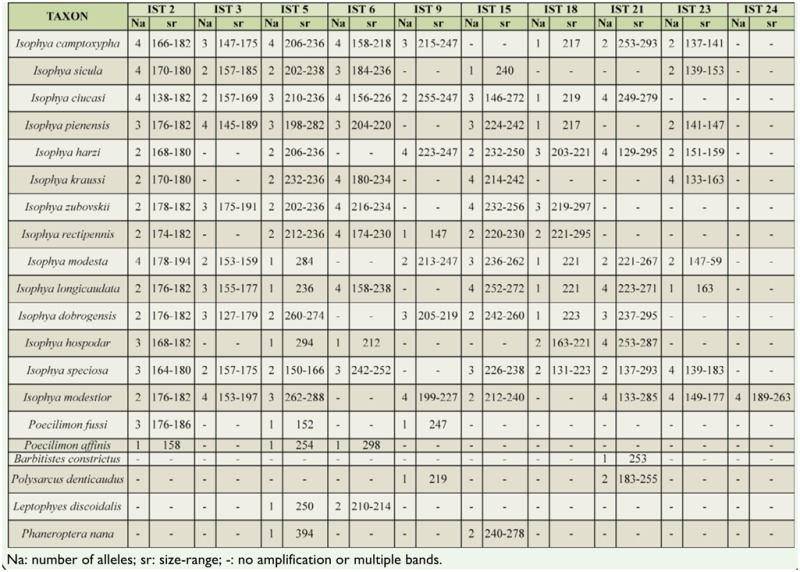
Cross-amplification of the microsatellite loci in 20 species of the family Phaneropteridae.
